# Factors Predictive of Publication Among Medical Students Participating in School-Sponsored Research Programs

**DOI:** 10.7759/cureus.18176

**Published:** 2021-09-21

**Authors:** Sean M Parker, Linda C Vona-Davis, Malcolm D Mattes

**Affiliations:** 1 Department of Medical Education, West Virginia University, Morgantown, USA; 2 Office of Research and Graduate Education, West Virginia University, Morgantown, USA; 3 Department of Radiation Oncology, Rutgers Cancer Institute of New Jersey, New Brunswick, USA

**Keywords:** medical student, clinical research, publication, mentor, mentorship

## Abstract

Introduction: Publishing research is an important component of medical students’ career development and becoming a more competitive residency applicant. Many medical schools offer structured programs to enable students to participate in research during their preclinical and clinical years, but the majority of student-mentor partnerships do not culminate in publication across a variety of institutions and medical specialties. The primary objective of this study is to determine if any factors associated with mentee-mentor partnerships are predictive of publication from two school-sponsored research programs at a single US medical school.

Methods: The PubMed-indexed publications of all student-mentor pairings from a summer internship (after year 1 of medical school) or research elective (during year 4 of medical school) at a single institution from 2008 to 2018 were retrospectively reviewed. Student/mentor demographic information was associated with the probability of publication.

Results: A total of 124 students participated in the summer internship with 32 (26%) achieving publication. The publication was significantly more likely for students that were from highly ranked undergraduate institutions (p* *= 0.04; likelihood ratio (LR) = 5.788), were future Alpha Omega Alpha (AOA) members (p = 0.03; LR = 4.597), or worked with a mentor focused on clinical rather than basic science research (p = 0.02; LR = 5.662). Forty-four students participated in the fourth-year elective with 11 (25%) achieving publication. The publication was more likely if the student worked with a mentor without a Doctor of Medicine (MD)/Doctor of Osteopathic Medicine (DO) degree (p* *= 0.001; LR = 7.051), with a PhD degree (p* *= 0.002; LR = 7.820), or a mentor with prior publication(s) with prior mentee(s) (p* *= 0.03; LR = 5.368).

Conclusion: Only one-quarter of mentor-mentee research pairings resulted in publication, with student-related factors more predictive for publication from the internship and mentor-related factors more predictive of publication from the elective. Approaches to promote successful completion of medical student research projects should be considered to yield the greatest value from students’ work and strengthen the development of future physician-scientists.

## Introduction

Medical students in the United States often engage in research with faculty mentors, with the potential for both mentee and mentor to derive a variety of benefits from the experience [1-3]. A students’ knowledge of research methodology can contribute toward a better understanding of interpreting and critiquing medical literature so that scientific evidence can be applied appropriately to clinical practice [4]. Some students may develop an interest in becoming physician-scientists themselves and improving patient care through discovery. While a research experience has value in and of itself, seeing projects through to publication is an important determinant of its success. Independent of the effect of a publication on changing clinical practice, publications are a critical component of a student becoming a competitive applicant for many specialties in the residency match [5], and research productivity in medical school has been associated with increased research productivity in residency and thus better career placement, especially in cultivating academic physicians [6-11].

Many medical schools offer structured programs to enable students to participate in research during their preclinical and clinical years, but the majority of student-mentor partnerships do not culminate in publication across a variety of institutions and medical specialties [1-2, 11-13]. It is likely that not all student-mentor partnerships and projects have a similar probability of publication from the outset, but little is known about what factors may impact this probability. Having this knowledge could better inform students, mentors, and academic administrators on ways to optimize the value of similar research experiences in the future. The primary objective of this study is to determine if any factors related to the student, mentor, or project are predictive of publication in two research programs at a single US medical school.

## Materials and methods

Study setting and participants

This retrospective review included medical students paired individually with a research mentor through (1) a summer research internship or (2) a research elective, at a single institution from 2008 to 2018. This study only included MD candidates and excluded MD/PhD candidates since the latter is on a different track with a greater focus on research in their education. The research internship consists of an optional 10-week paid experience (funding from Dean’s office) during the summer following a student’s first-year curriculum where mentors were chosen individually by students or assigned based on their specialty of interest. The research elective consists of an optional one-month experience during the fourth year of medical school where mentors were chosen individually by students. Prior to starting both programs, students were expected to submit proposals (under the guidance of their mentor) detailing the hypothesis, objectives, background, methods, and IRB approval information for the project. Upon completion of the program, students were expected to formally present their research results via presentation at an institution-sponsored research event or regional/national meeting.

The primary endpoint of this study was the mentee and mentor collaboration resulting in a publication, whereas a secondary endpoint was to determine the factors associated with multiple publications. Each endpoint was evaluated separately for the summer internship and fourth-year research elective.

Demographics of students and faculty mentors were collected to determine characteristics associated with a higher likelihood of publication. The evaluated independent variables related to the mentors included research focus (basic science or clinical), type of degree, clinical/tenure track level, prior number of publications prior to collaboration, years of institutional employment prior to collaboration, gender (deduced by name and online pictures, including gender discordance from student), prior research mentee(s) through internship or elective, and prior publication with research mentee(s) through internship or elective.

The evaluated independent variables related to the students included gender, number of prior publications, and undergraduate institution ranking (based on US News and World Report 2020 college rankings) [14]. Individual students’ grades and standardized test scores were not available for this study; however, to distinguish high-performing medical students, Alpha Omega Alpha (AOA) membership was used as a surrogate, even though students do not actually become AOA members until later in medical school. The primary factor considered for AOA membership at this institution is having grades in the top quartile of one’s class during their first two years of medical school. For those students who meet these criteria, secondary factors that are considered include receiving at least two letters of recommendation for academic achievement, attaining a high Step 1 score, taking on leadership positions, and being productive in research.

Data collection

A list of student and mentor names and titles of their project for each research program were obtained from the directors of each research program. Data related to mentor and mentee demographics were collected via internet search using Google to identify the individual on the website of their current employer, which was typically the primary source of information. This was supplemented and cross-checked with any additional information available on LinkedIn, Google, or prior residency/employment websites. Mentors’ information was also frequently available from curriculum vitae posted online. There were minimal discrepancies between the information on the websites that were available. The National Center for Biotechnology Information (NCBI) PubMed database was used to collect publication information. A publication was accredited to a given student-mentor pairing if both were listed as co-authors on the publication and the title of the publication was related to the title of the project in the research program’s records. In order to account for surname changes due to marriage, the mentor’s publication record was reviewed for both the first name and surname of their female student mentees, and any suspected surname changes were confirmed using similar internet searches as above. If a collaboration resulted in a publication, then the student authorship number, total number of authors, publication journal, and that journal’s associated impact factor were also recorded. Publications in “predatory journals,” which commonly describe open-access medical journals that publish articles online with little or no peer review, low academic standards, and little credibility, were not excluded if listed in PubMed.

Statistical analyses

Associations between the above endpoints and independent variables were evaluated using the Chi-squared, Fisher’s exact, and Mann-Whitney U tests, where appropriate. Fisher’s exact test was used for comparisons in which 20% of the cells had expected frequencies less than five; if the contingency tables were large, the Freeman-Halton extension was used [15]. Statistical analysis was carried out using Statistical Package for Social Sciences version 24.0 (SPSS Inc., Chicago, IL). A p-value less than 0.05 was considered statistically significant.

## Results

Study participants and characteristics

After approval by the local institutional review board, data were collected on a total of 336 individual students and their mentors. It included 168 students, with 124 (74%) participating in the summer internship and 44 (26%) participating in the research elective as presented in Table 1. Seven students participated in both programs and were included in the analysis of each. Gender distribution among medical students in both programs was different. Medical school enrollment at this institution is 48% females, but twice as many male medical students participated in the summer program compared to females (66.9% versus 33.1%, respectively); however, the elective year had a similar gender distribution between males and females (52.3% versus 47.7%, respectively). A large majority of students who participated in the summer research program (83.1%) had no previous publication record prior to entering the internship program. Across programs, fewer than 25% of medical students became members of the AOA Medical Honor Society. Students ultimately pursued a wide variety of medical and surgical residency programs after graduating from medical school.

**Table 1:** Medical student demographics * Based on US News and World Report 2020 college rankings.

	Internship, n (%)	Elective, n (%)
Gender		
Male	83 (66.9%)	23 (52.3%)
Female	41 (33.1%)	21 (47.7%)
Undergraduate Ranking*		
1–50	17 (13.7%)	3 (6.8%)
51–150	24 (19.4%)	5 (11.4%)
151–300	48 (38.7%)	24 (54.6%)
Not Ranked	18 (14.5%)	8 (18.2%)
Unknown	17 (13.7%)	4 (9.1%)
Prior Publications		
0	103 (83.1%)	27 (61.4%)
1-2	18 (14.5%)	16 (36.4%)
3 or Greater	3 (2.4%)	1 (2.3%)
Alpha Omega Alpha Member		
Yes	23 (18.6%)	11 (25.0%)
No	100 (80.6%)	32 (72.7%)
Unknown	1 (0.8%)	1 (2.3%)
Residency Position		
Internal Medicine	19 (15.3%)	4 (9.1%)
General Surgery	11 (8.9%)	3 (6.8%)
Anesthesiology	9 (7.3%)	5 (11.4%)
Orthopedics	9 (7.3%)	3 (6.8%)
Ob/Gyn	8 (6.5%)	2 (4.6%)
Family Medicine	7 (5.6%)	2 (4.6%)
Diagnostic Radiology	5 (4.0%)	2 (4.6%)
Pediatrics	4 (3.2%)	4 (9.1%)
Ophthalmology	4 (3.2%)	3 (6.8%)
Plastic Surgery	3 (2.4%)	2 (4.6%)
Emergency Medicine	2 (1.6%)	3 (6.8%)
Pathology	2 (1.6%)	----
Neurology	2 (1.6%)	1 (2.3%)
Radiation Oncology	2 (1.6%)	1 (2.3%)
Otolaryngology	2 (1.6%)	1 (2.3%)
Dermatology	1 (0.8%)	3 (6.8%)
Vascular Surgery	1 (0.8%)	2 (4.6%)
Psychiatry	1 (0.8%)	1 (2.3%)
Internal Medicine/Pediatrics	1 (0.8%)	----
Interventional Radiology	1 (0.8%)	----
Physical Medicine & Rehab	1 (0.8%)	----
Urology	----	1 (2.3%)
To Be Determined	23 (18.5%)	----
Not Applicable	6 (4.8%)	----

The distribution and characteristics of mentors included in the study are presented in Table 2. More male mentors were available to medical students in both the internship (81.5%) and elective program (72.7%). In the internship program, students selected more tenure track mentors with a PhD for basic science projects (62.1%), but this was reversed in the elective program where the majority of mentors were on a clinical track (68.2%). A total of 72.7% of mentors in the elective program had not mentored a student in either program previously, and 86.4% had a mentee with no prior research publications.

**Table 2:** Mentor demographics

	Internship, n (%)	Elective, n (%)
Gender		
Male	101 (81.5%)	32 (72.7%)
Female	23 (18.5%)	12 (27.3%)
Degree		
MD	39 (31.5%)	30 (68.2%)
PhD	77 (62.1%)	10 (22.7%)
MD/PhD	8 (6.5%)	4 (9.1%)
Clinical/Tenure Track Level		
Professor	70 (56.5%)	17 (38.6%)
Associate Professor	27 (21.8%)	8 (18.2%)
Assistant Professor	25 (20.2%)	11 (25.0%)
Unknown or N/A	2 (1.6%)	8 (18.2%)
Years at Institution		
1 to 5	45 (36.3%)	18 (40.9%)
6 to 10	35 (28.2%)	11 (25.0%)
11 or Greater	33 (26.6%)	7 (15.9%)
Unknown	11 (8.9%)	8 (18.2%)
Research Focus		
Basic Science	72 (58.1%)	6 (13.6%)
Clinical	51 (41.1%)	35 (79.6%)
Unknown	1 (0.8%)	3 (6.8%)
Prior Mentor Publications		
0 to 10	30 (24.2%)	29 (65.9%)
11 to 30	45 (36.3%)	13 (29.6%)
31 to 50	28 (22.6%)	2 (4.6%)
51 or more	21 (16.9%)	----
Prior Research Mentee		
Yes	63 (50.8%)	12 (27.3%)
No	61 (49.2%)	32 (72.7%)
Prior Mentee With Publication		
Yes	44 (35.5%)	6 (13.6%)
No	80 (64.5%)	38 (86.4%)
Mentor/Student Gender Concordance		
Concordant	76 (61.3%)	27 (61.4%)
Discordant	48 (38.7%)	17 (38.6%)
Gender Concordance Category (Mentor/Student)		
Male/Male	68 (54.8%)	19 (43.2%)
Male/Female	33 (26.6%)	13 (29.6%)
Female/Male	15 (12.1%)	4 (9.1%)
Female/Female	8 (6.5%)	8 (18.2%)

Publication outcomes of medical students in school-sponsored programs

For the summer internship cohort (n = 124), 32 collaborations resulted in publication (26%), with a median time until publication of 21 months (IQR, 17-30 months). Seven students (22%) were the first authors on the publication’s byline, 12 students (38%) were the second authors, and 13 students (41%) were the third (or higher) authors. The median impact factor of the journal of publication was 1.8 (IQR, 1.4-3.3). Among the students that published, 11 (34%) had multiple publications with their respective mentor, among which five students (16%) had three publications with their mentor, and two students (6%) had four publications with their mentor. At the time of this analysis, 78 of the 124 students (63%) who participated in the summer internship had at least one publication (with any mentor).

For the research elective cohort (n = 44), 11 collaborations resulted in publication (25%), with a median time until publication of 12 months (IQR, 4-19). Two students (18%) were the first authors on the publication’s byline, three students (27%) were the second authors, and six students (55%) were the third (or higher) authors. The median impact factor of the journal of publication was 2.4 (IQR, 1.0-3.9). Among the students that published, eight (73%) had multiple publications with their respective mentor with five students (45%) attaining three publications. At the time of this analysis, 30 of the 44 students (68%) who participated in the research elective had at least one publication (with any mentor).

Factors predictive of student-mentor publication

Categorical and continuous variables associated with student-mentor publication for each cohort are displayed in Tables 3, 4, respectively. Publication was significantly more likely for students who participated in the internship if they studied at a more highly ranked undergraduate institution (p = 0.04), would become an AOA member (p = 0.03), and worked with a mentor focused on clinical rather than basic science research (p = 0.02). Publication was significantly more likely for students who participated in the elective if they worked with a mentor without a Doctor of Medicine (MD)/Doctor of Osteopathic Medicine (DO) degree (p = 0.001), with a PhD degree (p = 0.002), or who had publication(s) with prior mentee(s) (p = 0.03).

**Table 3:** Associations between categorical independent variables and the probability of a mentor-mentee relationship resulting in publication for students who participated in the internship or elective programs AOA, Alpha Omega Alpha.

	Internship			Elective		
	Publication (%)	No Publication (%)	p-value	Publication (%)	No Publication (%)	p-value
Student Gender						
Male	21 (66%)	62 (67%)	0.86	8 (73%)	15 (46%)	0.12
Female	11 (34%)	30 (33%)		3 (27%)	18 (55%)	
Student Undergraduate Tier						
1 (#1–50)	8 (36%)	9 (13%)	0.04	2 (25%)	1 (4%)	0.23
2 (#51–150)	6 (27%)	18 (27%)		1 (13%)	4 (17%)	
3 (#151–300)	8 (36%)	40 (60%)		5 (63%)	19 (79%)	
Student Prior Publications						
0	27 (84%)	76 (83%)	0.23	6 (55%)	21 (64%)	0.79
1–2	4 (13%)	14 (15%)		5 (46%)	11 (33%)	
3 or More	3 (3%)	2 (2%)		0 (0%)	1 (3%)	
Student AOA Member						
Yes	10 (32%)	13 (14%)	0.03	4 (36%)	7 (22%)	0.28
No	21 (67%)	79 (86%)		7 (64%)	25 (78%)	
Mentor Gender						
Male	28 (88%)	73 (79%)	0.31	9 (82%)	23 (70%)	0.70
Female	4 (12%)	19 (21%)		2 (18%)	10 (30%)	
Mentor With MD/DO Degree						
Yes	12 (38%)	35 (38%)	0.96	4 (36%)	30 (91%)	0.001
No	20 (62%)	57 (62%)		7 (64%)	3 (9%)	
Mentor With a PhD Degree						
Yes	22 (69%)	63 (69%)	0.98	8 (73%)	6 (18%)	0.002
No	10 (31%)	29 (31%)		3 (27%)	27 (82%)	
Mentor Clinical/Tenure Level						
Assistant Professor	4 (13%)	21 (23%)	0.39	2 (22%)	9 (33%)	0.88
Associate Professor	7 (22%)	20 (22%)		2 (22%)	6 (22%)	
Professor	21 (66%)	49 (54%)		5 (56%)	12 (44%)	
Mentor Research Focus						
Basic Science	13 (41%)	59 (65%)	0.02	1 (9%)	5 (17%)	0.48
Clinical	19 (59%)	32 (35%)		10 (91%)	25 (83%)	
Mentor Had Prior Mentee(s)						
Yes	19 (59%)	44 (48%)	0.26	5 (46%)	7 (21%)	0.12
No	13 (41%)	48 (52%)		6 (55%)	26 (79%)	
Mentor Had Prior Publication(s) With Mentee(s)						
Yes	14 (44%)	30 (33%)	0.26	4 (36%)	2 (6%)	0.03
No	18 (56%)	62 (67%)		7 (64%)	31 (94%)	
Mentor/Student Gender Concordance						
Yes	17 (53%)	59 (64%)	0.27	8 (73%)	19 (58%)	0.30
No	15 (47%)	33 (36%)		3 (27%)	14 (42%)	

**Table 4:** Associations between continuous independent variables and the probability of a mentor-mentee relationship resulting in publication for students who participated in the internship or elective programs IQR, Interquartile range.

	Internship			Elective		
	Publication (IQR)	No Publication (IQR)	p-value	Publication (IQR)	No Publication (IQR)	p-value
Mentor Years at Institution	6 (4–11)	7 (4–12)	0.58	6 (5–10)	5 (4–8)	0.62
Prior Mentor Publications	19 (15–41)	23 (10–42)	0.80	10 (4–21)	4 (2–13)	0.07
Mentor Publications Within 3 Years	10 (6–16)	10 (5–15)	0.66	5 (3–8)	3 (0–7)	0.06

Categorical and continuous variables associated with multiple mentor-mentee publications for both internship and elective cohorts are displayed in Tables 5, 6, respectively. Multiple publications were significantly more likely for students who participated in the internship if they worked with a mentor focused on clinical rather than basic science research (p = 0.050). Multiple publications were significantly more likely for students who participated in the elective if the student had published previously (p = 0.045).

**Table 5:** Associations between categorical independent variables and the probability of a mentor-mentee relationship resulting in multiple publications for students participating in the internship or elective programs AOA, Alpha Omega Alpha.

	Internship			Elective		
	Multiple Publications (%)	No Multiple Publication (%)	p-value	Multiple Publications (%)	No Multiple Publication (%)	p-value
Student Gender						
Male	8 (73%)	75 (66%)	1.00	6 (75%)	17 (47%)	0.15
Female	3 (27%)	38 (34%)		2 (25%)	19 (53%)	
Student Undergraduate Tier						
1 (#1–50)	3 (38%)	14 (17%)	0.37	2 (40%)	1 (4%)	0.07
2 (#51–150)	2 (25%)	22 (27%)		0 (0%)	5 (19%)	
3 (#151–300)	3 (38%)	45 (56%)		3 (60%)	21 (78%)	
Student Prior Publications						
0	10 (91%)	93 (82%)	0.14	2 (25%)	25 (69%)	0.045
1–2	0 (0%)	18 (16%)		6 (75%)	10 (28%)	
3 or More	1 (9%)	2 (2%)		0 (0%)	1 (3%)	
Student AOA Member						
Yes	3 (27%)	20 (18%)	0.43	3 (38%)	8 (23%)	0.33
No	8 (73%)	92 (82%)		5 (63%)	27 (77%)	
Mentor Gender						
Male	9 (82%)	92 (81%)	1.00	7 (88%)	25 (69%)	0.29
Female	2 (18%)	21 (19%)		1 (12%)	11 (31%)	
Mentor With MD/DO Degree						
Yes	5 (46%)	42 (37%)	0.75	4 (50%)	30 (83%)	0.06
No	6 (54%)	71 (63%)		4 (50%)	6 (17%)	
Mentor With PhD Degree						
Yes	8 (73%)	77 (68%)	1.00	5 (63%)	9 (25%)	0.05
No	3 (27%)	36 (32%)		3 (38%)	27 (75%)	
Mentor Clinical/Tenure Level						
Assistant Professor	2 (18%)	23 (21%)	1.00	0 (0%)	11 (37%)	0.13
Associate Professor	2 (18%)	25 (23%)		1 (17%)	7 (23%)	
Professor	7 (64%)	63 (52%)		5 (83%)	12 (40%)	
Mentor Research Focus						
Basic Science	3 (27%)	69 (62%)	0.050	0 (0%)	6 (18%)	0.25
Clinical	8 (73%)	43 (38%)		8 (100%)	27 (82%)	
Mentor Had Prior Mentee(s)						
Yes	8 (73%)	55 (49%)	0.13	3 (38%)	9 (25%)	0.38
No	3 (27%)	58 (51%)		5 (63%)	27 (75%)	
Mentor Had Prior Publication(s) With Mentee(s)						
Yes	6 (55%)	38 (34%)	0.20	3 (38%)	3 (8%)	0.06
No	5 (45%)	75 (66%)		5 (63%)	33 (92%)	
Mentor/Student Gender Concordance						
Yes	6 (55%)	70 (62%)	0.75	5 (63%)	22 (61%)	0.64
No	5 (46%)	43 (38%)		3 (38%)	14 (39%)	

**Table 6:** Associations between continuous independent variables and the probability of a mentor-mentee relationship resulting in multiple publications for students participating in the internship or elective programs IQR, Interquartile range.

	Internship			Elective		
	Multiple Publication (IQR)	No Multiple Publication (IQR)	p-value	Multiple Publication (IQR)	No Multiple Publication (IQR)	p-value
Mentor Years at Institution	6 (5–10)	7 (4–12)	0.63	9 (5–13)	5 (4–8)	0.16
Prior Mentor Publications	16 (29–57)	22 (11–42)	0.28	10 (5–19)	4 (2–17)	0.19
Mentor Publications Within 3 Years	9 (5–16)	11 (5–15)	0.90	6 (3–8)	3 (0–7)	0.09

## Discussion

Research is a critical component of medical education, both in the context of practicing evidence-based medicine and for the purpose of scholarship and discovery. As such, opportunities for students to pursue mentored research projects are common at many medical schools [1,12,13] as well as through many national specialty societies hoping to promote greater academic interest in their field [11,16]. As in prior studies evaluating general research programs for all medical students regardless of their specialty of interest (or the specialty of their mentor), we found that approximately one in four mentor-mentee research pairings resulted in a manuscript publication in a PubMed-indexed medical journal, and two-thirds of participants published an article on any topic in their subsequent careers. This is comparable to what has been reported for a similar program at other institutions [2,11,17] and suggests that participation in any organized research program is associated with the pursuit of future research [16,18]. Our findings are unique in reporting outcomes of two different medical student research experiences at a single institution and in describing factors associated with publication.

Interestingly, student-related factors were most predictive of publication from summer internship projects, and mentor-related factors were most predictive of publication of fourth-year elective projects. It is perhaps not surprising that early in medical school, students who attended a more highly ranked college, or who have higher grades (as suggested by future AOA membership), would also be more successful in their research projects [19]. Attending a more highly ranked college may be associated with prior opportunities and exposure to research leading to a better understanding of how to perform in a research setting during medical school. Or alternatively, since most publications did not occur for many months afterward the research period, a student’s ability to merit co-authorship may have more to do with sustained engagement in the project even while balancing it with their medical school course work before and after the research period.

The type of project pursued is another important factor, with clinical projects typically being less time-consuming and perhaps easier to sustain during the school year than basic science laboratory work. Furthermore, although authorship criteria are relatively standardized across medical and biomedical journals, mentors’ views on what merits authorship may vary. For instance, it is possible that basic scientists may be more rigorous than their clinical counterparts in their authorship requirements since the former is perhaps more used to engaging MS or PhD degree candidates for whom publishing a manuscript is a component of earning their degree. Notably, predictors of a more prestigious first-author publication were also not assessed in this study due to the relatively small number of them and because the faculty mentors’ criteria to make a student first author are likely to vary substantially from one project to another based on the type of project, number and type of other people involved in it, and the relative contributions of each person. Unexpectedly, prior publications from the student or mentor did not impact the probability of publication from the pairing for the internship program.

The factors associated with publication in the fourth-year student elective program differed, likely related to the type of student who pursues this very different experience. Elective projects were published more quickly than internship projects and eight out of 11 students with publications in this cohort had multiple publications with their elective mentor. Both of these findings suggest students may have had pre-existing projects with mentors and completed them during the elective period. On the other hand, new elective projects with a clinical mentor, who a student perhaps met during the third- and fourth-year clinical rotations rather than selected specifically for research purposes, were less likely to result in publication. This may account for the fact that publication was more common with mentors with a PhD than with an MD or DO degree in the elective program. For instance, five physicians who served as elective research mentors had zero publications, including one mentor who worked with four students, and it is not surprising that none of these pairings resulted in a student publication. Unfortunately, some students may participate in the fourth-year elective for purposes other than research, like having free time for residency interviews. In general, we believe that if elective programs like this are going to be meaningful for students, there needs to be a more structured program with goals and expectations as well as significant vetting of experienced mentors who have a track record of publications, particularly with students they have worked with in the past. These types of programs have been implemented at some medical schools [1,13]. Other support structures like a mentor development program may also be worthwhile to help generate a larger pool of high-quality mentors to best serve students’ needs. Otherwise, students would be better served on any clinical rotation that the research elective took the place of. Clearly, clinical mentors and clinical projects can work well for students as shown in the internship data, and perhaps more clinicians need to promote their availability as a mentor during the summer internship when more basic science projects were pursued, despite more clinical projects being published.

There are several limitations to this study. Most importantly, it was conducted at a single institution, which is not among the top 40 US medical schools with the highest National Institute of Health research funding. Factors associated with publication for these types of medical student research programs may differ at more robust research institutions [20]. Furthermore, being a public medical school, the majority of students were from the same state or received an undergraduate degree at this state university and may have more homogeneous backgrounds than at some private medical schools, despite the wide variety of specialties that the students ultimately pursued. Also, PubMed was the only database searched, and it is possible that medical students’ articles were published in non-indexed journals or potentially using a different name.

Likewise, online searches to obtain demographic information has limitations for certain variables. For instance, while we believe that most surname changes for female students were captured (particularly related to publications with their mentor), some may not have been. Also, while the variables clinical/tenure track level and years of institutional employment provide some information on how advanced each faculty member was in their academic career, we could not determine the actual length of each faculty members’ academic career due to inability to obtain reliable prior employment history. There are also potentially other variables related to the mentor-mentee relationship and the extent of students’ contribution to the research that may have influenced publication and may have been better assessed using qualitative approaches that were not feasible in this retrospective study. Finally, there were only fewer students who participated in the research elective compared to the summer internship, which limits the power of our analysis for the elective cohort.

## Conclusions

The findings from this study may assist medical student administrators in looking critically at the research programs that they offer to their students and provide useful information to students and mentors as they select a research collaboration. Publication after the summer internship was more likely for students that were from highly ranked undergraduate institutions, were future AOA members, or worked with a mentor focused on clinical rather than basic science research. Publication after the research elective was more likely if the student worked with a mentor without an MD/DO degree, with a PhD degree, or a mentor with prior publication(s) with prior mentee(s).
